# Genetic epidemiology and Mendelian randomization for informing disease therapeutics: Conceptual and methodological challenges

**DOI:** 10.1371/journal.pgen.1006944

**Published:** 2017-10-05

**Authors:** Lavinia Paternoster, Kate Tilling, George Davey Smith

**Affiliations:** Medical Research Council Integrative Epidemiology Unit, University of Bristol, Bristol, United Kingdom; Stanford University School of Medicine, UNITED STATES

## Abstract

The past decade has been proclaimed as a hugely successful era of gene discovery through the high yields of many genome-wide association studies (GWAS). However, much of the perceived benefit of such discoveries lies in the promise that the identification of genes that influence disease would directly translate into the identification of potential therapeutic targets, but this has yet to be realized at a level reflecting expectation. One reason for this, we suggest, is that GWAS, to date, have generally not focused on phenotypes that directly relate to the progression of disease and thus speak to disease treatment.

A valuable proposed outcome of the GWAS era is the identification of novel therapeutic targets [1–4]. As of 3 April 2017, the GWAS Catalog contained 2,854 publications and 33,674 unique SNP-trait associations [5]. The large majority of these studies investigate genetic variation related to the presence (or occurrence) of disease. Such variants, though they may be informative for the prevention of disease, have unclear utility in informing disease treatment. If variants implicate etiological mechanisms of import for disease onset but sometimes of little relevance to disease progression, then the use of case/control GWAS as evidence to inform disease treatment–related drug discovery could be misleading. As an obvious example, consider GWAS of lung cancer. The lead variants identified in such GWAS tag a locus related to the heaviness of cigarette smoking [6], supporting the overwhelming evidence that smoking causes lung cancer. However, the cessation of smoking is hardly an efficacious treatment strategy after the onset of disease, although not smoking is a highly effective means of very substantially reducing the risk of developing lung cancer in the first place. Examples of factors causing both disease incidence and disease progression exist—for example, low-density lipoprotein (LDL) cholesterol levels clearly influence the risk of initial coronary events, and lowering LDL cholesterol reduces the risk of subsequent events. However, it is not necessarily the case that risk factors will influence both disease onset and disease progression—for example, a recent GWAS of Crohn disease observed independent genetic variants for the risk of onset and progression [7] and reported a negative genetic correlation (estimated through linkage disequilibrium [LD] score regression) between occurrence and progression, although this was imprecisely estimated. It is indeed possible that, in some cases, the effects of a particular exposure on the initiation and prognosis of disease could be in opposite directions, as has been suggested with respect to folate intake and colon cancer [8].

Few heritability studies of disease progression have been conducted. But there is evidence that disease progression phenotypes can have appreciable heritability (e.g., h^2^ = 74%–76% for progression of lumbar disc degeneration [9] and high inter-sibling concordance for multiple sclerosis progression [10]). In contrast to the large body of research on the genetic risk of disease incidence, only a small proportion of GWAS (approximately 8% of associations curated in the GWAS Catalog [*p* < 1 x 10^−5^]) have attempted to identify variants associated with disease progression or severity, and those that have are mostly small (90% have *n* < 5,000). This is most likely due to a research focus on mechanisms of the underlying causes of disease, as well as the limitation of available disease progression data, partly because measures of progression are harder to define and collect than more straightforward case/control phenotypes. But there is clearly interest in the GWAS field to conduct studies of progression, and so we expect to see an upward trend (in number and sample size) of such studies in the coming years, similar to that already seen for GWAS of disease occurrence. Investigating disease progression as a trait offers a considerable opportunity for identifying treatment targets and informing therapeutics, but it also introduces several important complications that have had little formal discussion in the literature and have not been addressed in many of the existing disease progression studies. A key problem, which we will discuss in more detail, is the issue of the potential introduction of collider bias when studying a selected (i.e., case-only) group of individuals.

GWAS are now routinely being used to help strengthen causal inference with respect to observational associations between exposures and disease by using Mendelian randomization (MR) (see Box 1) [11, 12]. With its emphasis on causality, it is important to appreciate that the challenges we present here also apply to MR. To date, few studies have used MR to identify factors influencing disease progression. In S1 Table, we summarize the 28 MR studies of progression that we identified in a systematic search. Only 1 of these studies [13] acknowledged the issue of the potential introduction of confounding through collider bias; interestingly, this was the first of these studies to be published.

**Box 1. Mendelian randomization.**

Mendelian randomization (MR) is an approach that uses genetic variation to improve causal inference in observational studies. A genetic variant associated with the exposure of interest (genetic instrument) is used to test the causal relationship between exposure and outcome (Fig 1). If there is an association between the genetic instrument and the outcome, then there is assumed to be a causal relationship because, unlike in the observational association, the genetic variant is not subject to issues of reverse causation and/or confounding. Assumptions of MR include the following [14]:

1. The genetic instrument is associated with the exposure of interest.
2. The genetic instrument is independent of factors that confound the association of the exposure and the outcome.
3. The genetic instrument is independent of the outcome, given the exposure and the confounders.

The method has been widely applied in the investigation of exposures that increase the risk of disease [15], both within single studies and in a 2-sample framework based on summary data, generally from large-scale genome wide association study (GWAS) consortia [16]. Such studies have demonstrated evidence of causal relationships (e.g., for obesity, blood pressure, and smoking with an increased risk of coronary heart disease [CHD] [17–19]), a lack of causal relationships (e.g., for C-reactive protein relationship with CHD, diabetes, and cancer [20–22]), debunking supposed protective behaviors (such as the beneficial effects of moderate alcohol intake on CHD risk [23]), and predicting randomized controlled trial successes and failures [24].

The emphasis on causality in a MR study has led to the acknowledgment within the field that they are also likely to have great value in suggesting what are likely to be successful interventions for the treatment of disease [25,26]. However, there are particular aspects of the study of disease prognosis that limit the applicability of MR.

**Fig 1:**
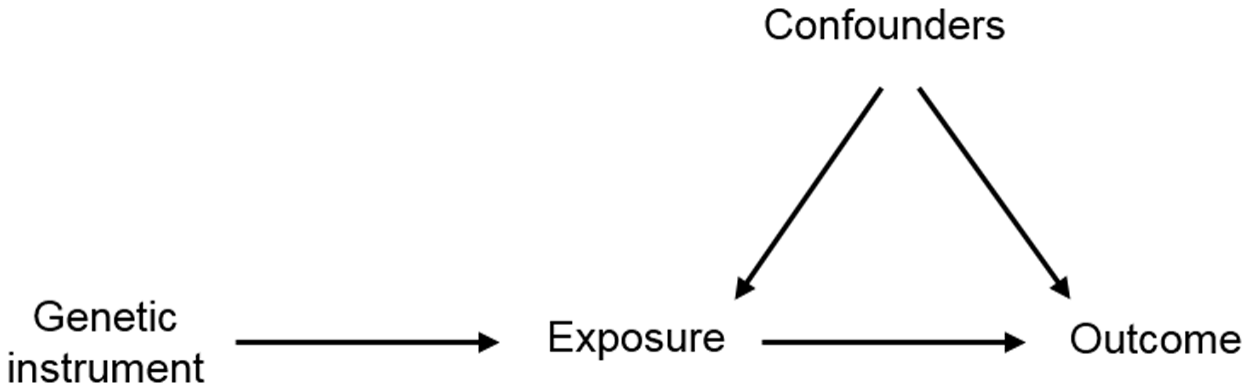
DAG of Mendelian randomization method. Abbreviation: DAG, directed acyclic graph.

## Challenges for genetic and MR studies of disease progression

### Collider bias

**Fig 2:**
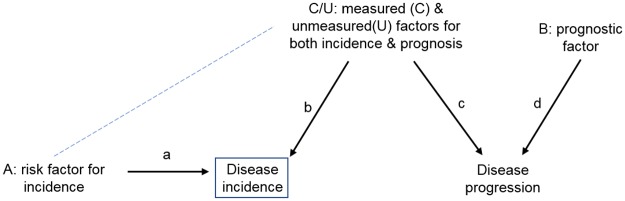
DAG demonstrating the issue of collider bias in studies with participants selected according to disease status. In this situation, collider bias can induce an association (dashed line) between any factors (A, C, and U) that affect disease incidence (or other study selection criteria). When 1 or more of these factors are also associated with disease progression (C, U), a path is opened up from A to disease progression through the induced association. If A is a genetic risk factor, it can appear that there is an association between genetic risk factor A and disease progression only because of the induced association with C or U. If C is measured and can be adjusted for, then the induced association is blocked, but unmeasured U cannot be adjusted for in the analysis. Only when the genetic risk factor for progression is not also a risk factor for incidence (i.e., B) will it not be affected by selection bias. The arrows in Figure 2 show causal paths between variables—e.g., that variable A causes disease incidence. A collider is a variable which has 2 paths entering it, e.g., disease incidence. A path is blocked by a collider—i.e., the path from A to disease progression is blocked by disease incidence. If a collider is conditioned on, then that path is unblocked—i.e., if disease incidence is conditioned upon, then the path from A to disease progression becomes unblocked (i.e., collider bias may occur). Abbreviation: DAG, directed acyclic graph.

Collider bias is a fundamental issue in progression studies [27]. Fig 2 shows an example causal diagram (or directed acyclic graph [DAG]) depicting the causes of disease incidence and progression. In this DAG, disease incidence is a collider because the paths from the risk factor for incidence (A) and the measured (C) and unmeasured (U) factors for both incidence and prognosis (C/U) collide at the disease incidence variable—i.e., A and C/U both cause disease. A collider blocks a path, so in this diagram, there is no path from A to C/U because the only path is blocked by disease incidence (the collider). However, conditioning on a collider opens the path (dashed line), which can then induce collider bias because there is then a noncausal path from A to C/U and onwards to disease progression. Conditioning on the collider can occur by stratifying on the variable or by adjusting for it in statistical analyses.

When a study group is selected on certain characteristics (e.g., being cases for a particular disease), this can introduce associations between all independent risk factors for these characteristics. For example, in a study of coronary heart disease (CHD) progression in which only CHD cases are selected for inclusion, there will be associations induced between all CHD risk factors (genetic and nongenetic) amongst the study individuals. Therefore, in a genetic study of progression within these cases, collider bias will induce spurious associations between genetic variants and progression (provided that at least 1 other factor influences both incidence and progression) [28]. Similarly, in an MR study of progression within these cases, the assumption that “the genetic instrument is independent of factors that confound the association of the exposure and the outcome” (assumption 2, Box 1) would be violated (see Box 2). Selection on case status does not automatically lead to bias: the presence, magnitude, and direction of the bias depend on the exact nature of the combined effects of the variables on disease status and the relationships between the variables.

**Box 2. Collider bias in Mendelian randomization.**

**Fig 3:**
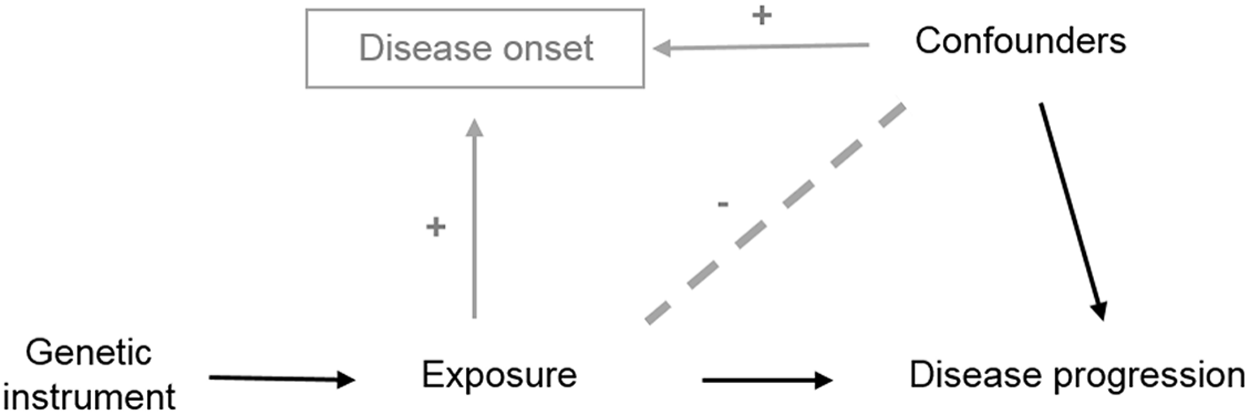
DAG to demonstrate how the introduction of collider bias through the selection of cases (grey paths) can impact an MR analysis between an exposure and disease progression as an outcome. Associations are induced because SNP causes disease (via exposure), and thus conditioning on disease induces an association between all variables causing disease. In a model not adjusting for exposure (e.g., relating SNP to progression), there is an association between SNP and the confounders, which biases the SNP-progression association. Abbreviations: DAG, direct acyclic graph; MR, Mendelian randomization.

Collider bias is an issue in Mendelian randomization (MR) of progression because, for any exposure that causes the onset of disease, the genetic instruments for that exposure may, amongst cases, be associated with other risk factors for onset, and so the association between the genetic variant and progression may be subject to confounding by these factors (Fig 3). Although this is true for single variants, the combination of variants into a polygenic score may serve to increase this effect [29].

We investigated the bias due to studying cases by only using a simple simulation study (Table 1). We simulated the situation depicted in Fig 2 with both a measured (C) and an unmeasured (U) confounder of disease incidence and progression. We simulated situations with low, moderate, high, and strong confounding. Collider bias has somewhat different implications for 2 underlying biological mechanisms. There is 1 (as depicted in Fig 2) in which risk factor A causes disease incidence, but A does not cause disease progression. In this scenario, studying only cases introduces collider bias, which induces an association between A and C and thus results in an induced association between A and disease progression in the study sample (Table 1). The bias in the estimated effect of A on disease progression increases as the degree of unmeasured confounding of disease incidence and progression increases (i.e., the degree to which there are common factors that influence disease onset and progression), with the proportion of 95% confidence intervals including the true effect of zero, falling from 90% (low confounding) to 35% (strongest confounding). The second scenario is one in which risk factor C causes both disease incidence and progression (Fig 2). Collider bias is again induced by studying only cases, and here it biases the estimated effect of C on progression towards the null (Table 1). Again, the bias increases as the degree of confounding of incidence and progression increases.

**Table 1:** Estimated effects of the risk factor for incidence only (A) and the risk factor for incidence and progression (C) from Fig 2 under different degrees of unmeasured confounding of incidence and progression.

	Degree of confounding by unmeasured confounder(s) (U)			
Simulated scenario	Low	Moderate	High	Strong
	OR for disease = 1.5	OR for disease = 2	OR for disease = 2.5	OR for disease = 3
	Beta for progression = 0.5	Beta for progression = 0.8	Beta for progression = 1	Beta for progression = 1.5
**Apparent effect of A on progression** (regression coefficient, SE) True effect = 0	−0.01 (0.01)	−0.02 (0.02)	−0.03 (0.02)	−0.06 (0.03)
**Percentage of 95% CI including 0**	90%	78%	66%	35%
**Apparent effect of C on progression** (regression coefficient, SE) True effect = 0.1	0.10 (0.01)	0.08 (0.01)	0.07 (0.01)	0.04 (0.02)
**Proportion of 95% CI including 0.1**	72%	35%	18%	1%

Each cell represents results from 500 simulations with a sample size of 50,000.

Uppercase letters refer to factors in Fig 2, lowercase letters refer to effect sizes of paths in Fig 2.

In all scenarios the OR for A and C for disease incidence are 1.3, and the MAF for genetic variants A is 0.2.

C and the U are standard normal variables, disease is a binary variable (with prevalence of approximately 0.2), and prognosis is a normally distributed variable.

Abbreviations: A, risk factor for incidence; C, measured factor for incidence and progression; MAF, minor allele frequency; OR, odds ratio; SE, standard error; U, unmeasured factor for incidence and progression.

This collider bias can lead to either an over- or under-identification of genetic risk factors for progression, depending on the direction of the relationships between the risk factors and disease onset. Collider bias should always be properly considered, and a number of things can be done to mitigate this potential bias.

1. Check for an association between the genetic variant and disease incidence in any study of disease progression. When a variant is identified as being associated with progression, the association between this variant and disease incidence (or other selection criteria) should also be reported. This can indicate whether there is any potential for collider bias.
2. Check for associations between the genetic variant and potential confounders in the study sample (that are not present in overall population)—such associations might indicate that both the genetic variant and confounders influence disease incidence [30].
3. If there are associations between a genetic variant and potential confounders of disease incidence and progression, then adjusting for such confounders will mitigate the problem. However, investigators should be aware that, as with any study of traditional risk factors, unmeasured confounding and measurement error in assessed confounders will remain an issue.
4. If certain parameters are known (such as the prevalence of disease and the effects of the genetic and potential confounders on disease onset), then it is possible to estimate the induced bias and so potentially correct for it using analytical formulae [28] or inverse probability weighting.

It is an important aside to note that, whilst disease incidence and diagnosis are the particular selection criteria of concern in the context of a progression study, any factor that relates to the selection of study participants can result in collider bias [27]. Therefore, any study in which the participants are not a random selection of the population can suffer from an induced association between genetic variants and factors that are independent in the underlying population.

### Confounding with disease stage at baseline

Studies of progression should be carefully designed so that it is true “progression” that is the outcome. Under some situations, disease detection (and hence the position of individuals along the disease progression timeline at diagnosis) may be associated with other factors (e.g., smoking could be related to age at onset). For example, suppose that older people were more likely to take part in a screening program because national screening programs often have a lower age limit. Thus, older people with cancer would tend to have their cancer detected earlier (by screening) and thus present with less advanced cancer, whereas younger people with cancer might present with symptomatic (more advanced) cancer. In a study of people with this cancer, it would appear that age was a positive prognostic factor. However, if stage at study entry was assessed, then the association between age and stage could be examined and controlled for in the analysis. Ideally, the stage of disease at study entry should be independent of the genetic variants. Collider bias with factors such as age might violate this—if age and genetic variant both influence disease incidence, and age influences stage of disease at study entry, then in a case-only study, the genetic variant would appear to be associated with age and hence also with the stage of disease at study entry. In this example, this spurious correlation could be removed by adjusting for age—however, in practice, all the factors influencing the risk of disease occurrence will not be known.

### Measurement of progression

GWAS and MR typically use a single measure of either a continuous (e.g., blood pressure at age 60) or a binary (e.g., occurrence of a myocardial infarction by age 60) outcome. In a study of progression, the outcome may be more complex: time to cancer recurrence; survival time; the accumulation of disability over a 20-year period; or recurrence-free survival time. For these outcomes, a more sophisticated analysis may be required. When survival is the outcome of interest, disease-specific as well as all-cause mortality should be investigated, and disease-specific survival analysis will need to account for censoring (missing follow-up data) due to death from other factors. Similarly, GWAS analysis methods for trajectories will be required for studies in which the outcome is a repeated quantitative measure (e.g., the progression of disability in multiple sclerosis). We have developed a methodology for GWAS of trajectories [31,32], and methods for MR in the context of survival analysis are available [33], but computational challenges remain, and further methodological development is much needed. In addition, to allow well-powered meta-analysis studies to be conducted, comparable measures of progression will need to be available across data sets.

### Availability of data

GWAS and MR of disease occurrence have had huge recent success, in no small part due to the availability of very large data sets. In order for GWAS and MR of progression to see the same success, there is a need for large-scale studies with both progression and genetic data. One potential source of such data is randomized controlled trials, which will have detailed follow-up of patients and often now collect DNA as a standard. Genome-wide genotyping of such resources is an important first step. The generation of valuable progression data for GWAS is likely to require large consortia collaboration (as has been the case for traditional GWAS). Therefore, the standardization of progression measures across a number of studies is also going to be important for this approach to reach its full potential.

If all of these issues are appropriately addressed, there is a huge opportunity for GWAS and MR of disease progression to identify potential new treatments [34]. Platforms such as MR-Base (www.MRbase.org) [35], which catalogs all available GWAS data for the simple implementation of MR, will make it possible to screen a wide array of modifiable risk factors and drug targets to prioritize those for evaluation as treatments for disease.

## Supporting information

S1 Table Mendelian randomization studies of progression. These were either known to us or identified through PubMed search (progression OR prognosis OR survival OR mortality) AND (Mendelian randomization OR Mendelian randomization, searched on 1 May 2017).
(XLSX)
